# Epidemiologic study of chronic hepatitis B virus infection in male volunteer blood donors in Karachi, Pakistan

**DOI:** 10.1186/1471-230X-5-26

**Published:** 2005-08-08

**Authors:** Saeed Akhtar, Muhammad Younus, Salman Adil, Farrukh Hassan, Sarffraz Hussain Jafri

**Affiliations:** 1 Department of Community Medicine and Behavioral Sciences, Faculty of Medicine, Kuwait University PO Box 24923, Safat 13110, Kuwait; 2 Department of Community Health Sciences, Aga Khan University, Karachi 74800, Pakistan; 3 Department of Pathology and Microbiology, Aga Khan University, Karachi 74800, Pakistan; 4 Husaini Blood Bank, Karachi, Pakistan

**Keywords:** hepatitis B virus, hepatitis B virus surface antigen, prevalence, risk factors, volunteer blood donors, Pakistan

## Abstract

**Background:**

The magnitude of chronic infection with hepatitis B virus (HBV) varies substantially between the countries. A better understanding of incidence and/ or prevalence of HBV infection and associated risk factors provides insight into the transmission of this infection in the community. The purpose of this investigation was to estimate the prevalence of and to identify the risk factors associated with chronic infection with HBV, as assessed by HBV surface antigen (HBsAg) positivity, in asymptomatic volunteer male blood donors in Karachi, Pakistan.

**Methods:**

Consecutive blood donations made at the two large blood banks between January 1, 1998 and December 31, 2002 were assessed to estimate the prevalence of HBsAg positivity. To evaluate the potential risk factors, a case-control study design was implemented; cases (HBsAg positives) and controls (HBsAg negatives), were recruited between October 15, 2001 and March 15, 2002. A pre-tested structured questionnaire was administered through trained interviewers to collect the data on hypothesized risk factors for HBV infection. Sera were tested for HBsAg using commercially available kits for enzyme linked Immunosorbant assay-III.

**Results:**

HBsAg prevalence in the male volunteer blood donors was 2.0 % (7048/351309). Multivariate logistic regression analysis showed that after adjusting for age and ethnicity, cases were significantly more likely than controls to have received dental treatment from un-qualified dental care provider (adjusted odds ratio (OR) = 9.8; 95% confidence interval (CI): 2.1, 46.1), have received 1–5 injections (adjusted OR = 3.3; 95% CI: 1.1, 9.6), more than 5 injections (adjusted OR = 1.4; 95% CI: 1.4, 12.7) during the last five years or have received injection through a glass syringe (adjusted OR = 9.4; 95% CI: 2.6, 34.3). Injury resulted in bleeding during shaving from barbers (adjusted OR = 2.3; 95% CI: 1.1, 4.8) was also significant predictor of HBsAg positivity.

**Conclusion:**

Prevalence of HBsAg positivity in the male volunteer blood donors in Karachi was 2%. Infection control measures in health-care settings including safe injection practices and proper sterilization techniques of medical instruments and education of barbers about the significance of sterilization of their instruments may reduce the burden of HBV infection in this and similar settings. There is also an urgent need of developing locally relevant guidelines for counseling and management of HBsAg positive blood donors.

## Background

In the absence of effective screening programs, hepatitis B virus (HBV) is responsible for a substantial proportion of cases of post-transfusion hepatitis, liver cirrhosis and hepatocellular carcinoma [1]. An estimated 2 billion people are infected with HBV worldwide, among them 350 millions are chronic carriers: hepatitis B surface antigen (HBsAg) positive [2]. HBsAg positivity in developed countries varies from 0.6 percent in Wales, England, to 1.2 percent in Texas, USA. However, higher prevalences of infection with HBV have been reported from various parts of the developing world including 3.5% in Gaza, Palestine [3], 1.6%–7.7 % in Brazil [4,5], 19.6 % in Egypt [6], and 2%–10 % from various parts of India [7].

Intravenous drug use, needle stick injuries, haemodialysis, tattooing and multiple sexual partners have been identified as common modes of HBV transmission in the developed world [8]. In many developing countries however, the relative contributions of various routes of HBV infection have not been defined in population-based studies. Due to a lack of universal and appropriate blood screening in these countries, the risk of post-transfusion HBV infection is still unknown. Parenteral routes implicated as the most likely factors for HBV transmission include un-sterilized needles and syringes in health-care settings [9-11], Moreover, in low socio-economic settings, horizontal transmissions of HBV through contact with infected family member have also been reported [12], but these findings are yet to be verified.

The national estimates for prevalence and/or incidence of HBV infection in Pakistan are unknown. However, studies in selected groups have shown variable prevalence of chronic infection with HBV as assessed by HBsAg positivity: 7% in health professionals [13], 2%–14% in blood donors [14-17]. Pre-employment screening revealed 2.6% HBsAg positivity among the healthy individuals in northern Pakistan [18]. Moreover, some hospital-based studies have revealed that 30% – 42% of the cases of chronic liver disease [19,20] and 78% of the cases of hepatocellular carcinoma [19] were positive for HBsAg.

Developed countries have been successful in reducing the risk of HBV spread by interrupting some of the known routes of HBV transmission and through mass HBV vaccinations. The vaccine against HBV infection is available in most of the developing world including Pakistan, but its high cost limits the widespread use. Recently, Pakistan initiated universal HBV vaccination for neonates through its expanded program of immunization with the assistance of Global Alliance for Vaccines and Immunization [2]. However, public health benefits of this initiative require some time to accrue as the program focuses on neonates only [10]. Therefore, a multi-prong approach needs to be undertaken to curtail the spread of HBV infection in Pakistan and perhaps other developing countries in the region.

Volunteer blood donors are generally regarded as a healthier segment of any community, as blood banks usually have strict selection criteria that helps identify, and consequently bleed healthy donors only [21]. The proportion of HBsAg positive donors and risk factor(s) associated with HBsAg positive status among these healthy individuals may reflect the magnitude of chronic HBV infection in the general population. Therefore, we conducted this study to estimate the prevalence of and to identify the risk factors associated with HBsAg positivity in asymptomatic male volunteer blood donors in Karachi.

## Methods

### Study design and setting

The study design, setting and data collection procedures have been described elsewhere [22], and we briefly outlined here. This study was conducted at two large blood banks (I and II) in Karachi. Blood bank I is located in a tertiary care teaching hospital in private sector and caters for the need of inpatients. Blood bank II belongs to a non-government organization (NGO) and caters the need of all those who need blood transfusion including patients of leukaemia, haemophilia, thalassaemia and other blood related diseases. Both the blood banks receive blood donations as replacements from volunteers and admit for screening apparently healthy individuals. Preliminary screening includes a personal interview with the donor and exclusion of those who admit to known risk factors for hepatitis B and C and human immunodeficiency virus infections.

HBsAg test results of consecutive blood donations made between January 1, 1998 and December 31, 2002 at blood bank I, and from January 1, 1999 to December 31, 2002 at blood bank II were available to assess the proportions of HBsAg positive donors. To evaluate the potential risk factors for HBsAg positivity, a case-control study design was implemented.

### Recruitment of cases and controls

Between October 15, 2001 and March 15, 2002, eligible blood donors aged 18–64 years, and HBsAg positive were defined as cases and those who were HBsAg negative were taken as controls. Study subjects were contacted after obtaining their addresses and phone numbers from records of respective blood banks. After explaining the objectives and potential risks/benefits of the study, subjects were invited to participate in the study.

### Data collection and serology

A pre-tested structured questionnaire was used to collect data from both cases and controls regarding demographic, and socioeconomic attributes, various potential parenteral exposures to blood or blood products and non-parentral modes of HBV transmission. Both the blood banks use commercially available enzyme-linked immunosorbant assay-III (ELISA) kits to detect HBsAg as a marker for chronic HBV infection. ELISA results are interpreted essentially following the instructions of the manufacturer. This study complied with the human subjects' protection requirement of the institutional ethics committee.

### Data analysis

Data were managed and analyzed using Epi-Info (version 6.04: Centres for Disease Control and Prevention, Atlanta, GA, USA) and SPSS (version 10.0: SPSS Inc., Chicago, IL, USA) respectively. Overall HBsAg prevalence and HBsAg prevalence by year and by blood bank was computed. Chi-square analysis for trend was carried out to assess the significance of the change in proportions of HBsAg positive donors over the entire study period. Descriptive statistics were computed for demographic variables for both cases and controls. To asses univariate associations between HBsAg positivity and hypothesized risk factors, odds ratios (ORs) and their corresponding 95% confidence intervals (CIs) were calculated using simple logistic regression analysis. A final set of independent risk factors for HBsAg positivity among the donors was derived by a backward stepwise logistic regression model. The risk factors significantly (P = 0.2) related with outcome on univariate analyses were considered for inclusion in the final model. After arriving at main effects model, plausible interaction terms were also evaluated for inclusion in the model. The adjusted ORs and their 95% CIs were obtained from final model and used for substantive interpretation of the model. The fit of the final model was assessed by the Hosmer-Lemeshow's goodness-of-fit test [23].

## Results

### HBsAg prevalence

The overall HBsAg prevalence in this study was 2.0% (7048/351309). HBsAg positivity among the donors at blood bank I was 1.0 % (782/75752), whereas, corresponding figure for the donors at blood bank II was 2.3% (6266/275557). Blood donations and proportions of HBsAg positive donors by blood bank and by year are given in Table 1. The proportions of HBsAg positive donors were consistently higher at blood bank II than blood bank I across the study period. Trend analysis revealed an overall significant (P < 0.001) increase in the proportions of HBsAg positive donors from 1998 to 2002 for combined data and for the data from blood bank I. However, for blood bank II there was significant (P < 0.001) downward trend in the proportions of HBsAg positive donors.

### Characteristics of cases and controls

In this study 64 cases and 260 controls were enrolled. About 81% of cases and 86% of controls were 35 years of age or less. Forty seven percent of cases and 61% of controls belonged to Mohajir ethnicity, almost representing the composition of general population of Karachi. Fifty three percent of cases and 61% of controls had 10 or more years of schooling and 45% of cases were ever-married compared to 56% of controls. The distributions of profession, household income, number of blood donations in the past, parenteral and non-parenteral factors considered are given in Table 2.

### Risk factor analysis

On univariate analyses, death of a family member due to liver disease, dental treatment received from a un-qualified provider, therapeutic injections received in the past, type of syringe used for therapeutic injections, intravenous infusions received in the past, bleeding during shaving from barbers and sexual intercourse with multiple partners were significantly (*P *≤ 0.05) associated with HBsAg positivity (Table 2).

Final multivariate logistic regression model revealed that cases were significantly more likely than controls to have received dental treatment from un-qualified provider (adjusted OR = 9.8; 5% CI: 2.1, 46.1). Also, cases compared to controls were significantly more likely to have received 1–5 injections (adjusted OR = 3.3; 5% CI: 1.1, 9.6) or >5 injections (adjusted OR = 1.4; 5% CI: 1.4, 12.7) during the last five years. Furthermore, cases were significantly more likely to have been injected medicine with a glass syringe by health-care providers (adjusted OR = 9.4; 5% CI: 2.6, 34.3). Bleeding during shaving from barbers was independently and significantly associated with HBsAg positivity (adjusted OR = 2.3; 5% CI: 1.1, 4.8) in this study. The Hosmer-Lemeshow goodness-of-fit test statistic showed a good fit for the final model (χ^2 ^= 7.82, *P *= 0.349) (Table 3).

## Discussion

The objectives of this study were to estimate the prevalence of and to identify the risk factors for chronic infection with HBV as assessed by the HBsAg positivity through ELISA-III among volunteer blood donors in Karachi. An overall 2% HBsAg prevalence among volunteer blood donors in this study was recorded, which is lower than the figures reported from some other high HBV prevalence developing countries including 3.4% in Georgia [24], 4.5% in Taiwan [25], and 5.8% in Indonesia [26]. With HBsAg prevalence of 2%, Pakistan falls into intermediate range of HBV infection suggesting a need for screening of pregnant women at minimum and universal childhood vaccination against HBV. However, a much lower prevalence of infection with HBV has been reported from some developed countries, for instance 0.8% in Australia [27] and 1.1% in Denmark [28], which reflect the efficacy of blood donor selection policies, effective screening programs and very low HBV prevalence in general population in these countries. Similar strategies may play a significant role in reducing post-transfusion HBV infection in Pakistan.

The proportions of HBsAg positive blood donors were consistently higher at blood bank II (2.3%) than at blood bank I (1.0%) across the study period. As mentioned earlier that blood bank I receives blood donations from the relatives/friends of the hospital in-patients only, and usually patients from middle and higher socioeconomic class utilize the services of this hospital. Being a subsidiary of an NGO, blood bank II caters the need of all patients requiring blood transfusion in the city including patients from public funded hospitals, which are generally attended by the lower or middle class patients. It has been well documented that HBV infection is more prevalent in low socioeconomic settings in Indonesia [29], or perhaps similar setting in Pakistan. Therefore, the difference in the HBsAg positivity may be attributed to the apparent difference in populations attending these two blood banks. Although trend analysis revealed significant changes over time (1998–2002) in proportions of donors positive for HBsAg at blood bank I, blood bank II and for combined data but these changes do not seem to be substantial. Therefore, given the large sample size and the observational design of the study, this statistically significant change in the proportions over time may be discounted. Nonetheless, further study is indicated to discern this pattern using detailed time series data.

We found that subjects with a history of dental treatment received from unqualified provider had an increased risk for HBsAg positivity; consistent with previous research [30]. Dental practice by unregistered practitioners is common in developing countries. These non-medical personals, besides their technical incompetence, do not properly sterilize their equipments and thereby transmit blood borne infections to their patients. Strict enforcement of legislation to ban such illegal practices may result in substantial reduction in HBV transmission in this and similar settings.

Therapeutic injections received during the past five years were significantly associated with HBsAg status in this study. HBV transmission through contaminated needles is well established [31]. In Pakistan, the proportion of injections per prescription is one of the highest compared to some other countries [32-34]. In developing countries, a large proportion of patients preferred injected medicines and considered them more efficacious than other routes of drug administration [32,34]. In addition to patient preference for injections, physicians' prescribing practices, their belief in better efficacy of injected drugs, direct observation of the prescribed therapy, patients demand and financial incentive have been reported as the reasons for increased frequency of injections in developing countries [35]. Therefore, interventions to improve injection safety and reduce injection overuse would have a substantial impact on the incidence of infection with HBV.

This study showed a strong association between use of glass syringe for therapeutic injection and HBsAg status. The use of glass syringes in administering therapeutic injections has been common in the past, and is still in practice in most of the low socioeconomic settings in developing countries. The 'boiling method' employed to sterilize glass syringes in most of the health facilities is in-adequate for complete sterilization. Therefore, these glass syringes act as a source for transmitting HBV and other blood borne pathogens [7,36-38]. Taking into account the hazards of glass syringes, their use should be banned by public health authorities in Pakistan and perhaps other developing countries in the region.

Bleeding during facial shaving from barbers was significantly associated with the outcome in this study. Facial shaving from barbers has been repeatedly documented as a risk factor for transmission of hepatitis B and C viruses in various countries [39-41]. Barbers in this part of the world are mostly un-aware of the transmission of blood borne pathogens through shaving tools [41]. The repeated use of potentially contaminated razors and other non-sterilized shaving tools most likely infect their clients with HBV and other blood-borne infections. Therefore, health education programs focusing on barbers' community may contribute to the reduction in HBV transmission in this and other similar settings.

Some limitations of this study need to be considered in interpreting the present findings. Volunteer blood donors are a low-risk healthier segment of any community [42], who are further screened for symptoms of various medical conditions at the blood banks. Hence, we expect a considerable 'healthy donor effect – relatively weaker associations of the risk factors with HBsAg positivity in this population. Furthermore, subjects with long-standing HBsAg positive status could be sicker with hepatocelluar carcinoma or cirrhosis, perhaps too sick to come to donate blood and resulting into underestimation of prevalence of HBsAg positivity. Recall bias – an inherent limitation of a case-control design, might have been introduced in measurement of some of the variables, especially when past histories of injections and intravenous infusions were explored [43]. However, to minimize recall bias, we asked injection and infusion history of different eras: six months to ten years. Any bias that might have occurred must be non-differential, thus yielded conservative estimates of observed relationships. For economic reasons we were unable to test for some other markers of active HBV infection such as HBV DNA and/or hepatitis B e antigen (HBeAg), and IgM anti-HBc – an indicator of early acute HBV infection,. Therefore, some donors in this category may have been missed since some patients with acute self-limited primary HBV infection never have detectable HBsAg in the blood [44]. However, considering the sample size in this study, we hope such misclassification to be minimal. In the case-control arm of the study, we did not test controls for anti-HBs (antibodies to HBsAg) to exclude those who might have resolved past infection, became immune and lacked potential to become case as defined in this study. Ascertainment of controls' serostatus may have non-differentially led to controls who are not susceptible to HBV infection due to previous infection or immunization, thereby biasing results toward the null. Future studies should take this aspect in to account. The proportion of female donors in the selected blood banks was quite low; therefore, excluded from the present analysis. Study in female blood donors might show a different set of risk factors.

## Conclusion

The prevalence of HBsAg positivity was 2% in male volunteer blood donors in this study. A history of dental treatment received from un-qualified provider, the number of therapeutic injection received during the past five years and use of glass syringe for therapeutic injections, bleeding during facial shaving from a barber were independent risk factors for HBsAg positivity in this study population. Therefore, educational intervention targeted on health-care professionals about the importance of infection control measures may include safe injection practices and proper sterilization of medical and dental instruments. Education of barbers about the significance of sterilization of their instruments may help in reducing the burden of community-acquired infection with HBV and other blood-borne pathogens in this and similar settings. Strict enforcement of legislation to ban un-qualified dental practitioners may further help curb the HBV spread. There is also an urgent need to develop locally relevant guidelines for further management of HBsAg positive donors. A cohort study of donors preferably on those who routinely volunteer should focus both on acute and well as chronic HBV infection to seek better estimate of the magnitude of the problem by incorporating relevant diagnostic tests.

## Competing interests

The author(s) declare that they have no competing interests.

## Authors' contributions

SA conceived and designed the study. SA and MY designed the study questionnaire. MY, SD, FH SHJ participated in the implementation of the study. SA and MY were responsible for data analysis and manuscript writing. All the authors read and approved the manuscript.

## Pre-publication history

The pre-publication history for this paper can be accessed here:



## Figures and Tables

**Table 1:** Total blood donations and proportions of hepatitis B virus (HBV) infected donors at two large blood banks in Karachi, 1998–2002

Blood bank 1				Blood bank 2			Blood banks combined		
		
Year	Total	HBV^+^	(%)^a^	Total	HBV^+^	(%)^b^	Total	HBV^+^	(%)^c^
1998**	15068	117	(0.8)	--		--	15068	117	(0.8)
1999	14856	72	(0.5)	62566	1530	(2.4)	77422	1602	(2.1)
2000	15525	95	(0.6)	66486	1566	(2.4)	82011	1661	(2.0)
2001	15084	230	(1.5)	74154	1654	(2.2)	89238	1884	(2.1)
2002	15219	268	(1.8)	72351	1516	(2.1)	87570	1784	(2.0)
									
Total	75752	782	(1.0)	75557	6266	(2.3)	351309	7048	(2.0)

**Data for 1998 were not available for blood bank 2^;a^χ^2^_trend _= 170.81 (*P *< 0.001); ^b^χ^2^_trend _= 21.28 (*P *< 0.001); ^c^χ^2^_trend _= 123.05 (*P *< 0.001)

**Table 2:** Univariate analysis of putative risk factors for hepatitis B virus infection in a case-control study of volunteer blood donors from two large blood banks, Karachi, Pakistan

Variables	Cases		Controls		OR†	95% CI^‡^
			
	n = 64	%	n = 260	%		
***Blood bank***						
Blood bank 1	38	(59.4)	118	(45.4)	1.0	-
Blood bank II	26	(40.6)	142	(54.6)	0.6	0.3–1.0

*Age (completed years)*						
15–25	23	(35.9)	139	(53.5)	1.0	-
26–35	29	(45.3)	86	(33.1)	2.0	1.1–3.8
> 36	12	(18.8)	35	(13.5)	2.1	0.9–5.6

Ethnicity						
Mohajir	30	(46.9)	158	(60.8)	1.0	-
Punjabi	8	(12.5)	45	(17.3)	0.9	0.4–2.2
Pathan	11	(17.2)	17	(6.5)	3.4	1.5–8.0
Sindhi	5	(7.8)	15	(5.8)	1.8	0.6–5.2
Baloch and others	10	(15.6)	25	(9.6)	2.1	0.9–4.8

Education (completed school years)						
>10	34	(53.1)	159	(61.2)	1.0	-
≤ 10	30	(46.9)	101	(38.8)	1.4	0.8–2.5

*Marital status*						
Never married	29	(45.3)	146	(56.2)	1.0	-
Ever married	35	(54.7)	114	(43.8)	1.5	0.9–2.7

*Profession*						
Un-employed	8	(12.5)	50	(19.2)	1.0	-
Self-employed	19	(29.7)	39	(15.0)	3.0	1.1 – 8.6
Service provider	37	(57.8)	171	(65.8)	1.4	0.6 – 3.4

*Household income (Pak Rs./mo)						
> 15000	11	(18.3)	33	(15.3)	1.0	-
8001–15000	16	(26.7)	71	(33.0)	0.7	0.3 – 1.8
5001–8000	9	(15.0)	50	(23.3)	0.5	0.2 – 1.6
≤ 5000	24	(40.0)	61	(28.4)	1.2	0.5 – 2.9

*Number of times donated blood*						
1	21	(32.8)	93	(35.8)	1.0	-
2	13	(20.3)	52	(20.0)	1.1	0.5–2.6
*>2*	30	(46.9)	115	(44.2)	1.2	0.6–2.3

*History of hospital admission*						
0	50	(78.1)	196	(75.4)	1.0	-
1	13	(20.3)	53	(20.4)	0.9	0.5–2.0
>1	1	(1.6)	11	(4.2)	0.4	0.1–2.8

*History of dental treatment*						
Never had dental treatment	31	(48.4)	183	(70.4)	1.0	-
Treated by dentist	24	(24.0)	73	(28.1)	1.9	1.1–3.5
Treated by others	9	(9.0)	4	(1.5)	13.2	3.9–45.8

*Last injection received (completed months)*						
Never received injection	5	(7.8)	63	(24.2)	1.0	-
≥ 6	34	(53.1)	123	(47.3)	3.5	1.3–9.3
< 6	25	(39.1)	74	(28.5)	4.3	1.5–11.8

*Injections received in last one year*						
0	27	(42.2)	167	(64.2)	1.0	-
1	8	(12.5)	19	(7.3)	2.6	1.0–6.5
2 – 5	19	(29.7)	49	(18.8)	2.4	1.2–4.7
≥ 6	10	(15.6)	25	(9.6)	2.5	1.1–5.7

*Injections received in five years*						
0	15	(23.4)	119	(45.8)	1.0	-
1–5	23	(35.9)	84	(32.3)	2.2	1.1–4.4
> 5	26	(40.6)	57	(21.9)	3.6	1.8–7.4

*Injections received in ten years*						
0	40	(62.5)	167	(64.2)	1.0	-
1–10	16	(16.0)	75	(28.8)	0.9	0.5–1.7
>10	8	(8.0)	18	(6.9)	1.9	0.7–4.6

*Type of syringe used for the last injection*						
Never had injection	8	(12.5)	92	(35.4)	1.0	-
Plastic syringe	31	(48.4)	131	(50.4)	2.7	1.1–6.2
Glass syringe	25	(39.1)	37	(14.2)	7.8	3.2–18.8

*IV infusion received from*						
Never had drip	36	(56.3)	156	(60.0)	1.0	-
Hospital	6	(9.4)	49	(18.8)	0.5	0.2–1.3
GP's and others	22	(34.4)	55	(21.2)	1.7	0.9–3.2

*IV infusion received in last one year*						
0	53	(82.8)	240	(92.3)	1.0	-
1	5	(7.8)	16	(6.2)	1.4	0.5–4.0
≥ 2	6	(9.4)	4	(1.5)	6.8	1.9–25.0

*IV infusion received in last five years*						
0	47	(73.4)	202	(77.7)	1.0	-
1–4	12	(18.8)	54	(20.8)	1.0	0.5–1.9
≥ 5	5	(7.8)	4	(1.5)	5.3	1.4–20.8

*IV infusions received in last ten years*						
0	42	(65.6)	171	(65.8)	1.0	-
1–4	15	(23.4)	79	(30.4)	0.8	0.5–1.5
≥ 5	7	(10.9)	10	(3.8)	2.9	1.0–8.0

*Ear pierced*						
No	62	(96.9)	248	(95.4)	1.0	-
Yes	2	(3.1)	12	(4.6)	0.7	0.2–3.1

*Tattooing*						
No	61	(95.3)	249	(95.8)	1.0	-
Yes	3	(4.7)	11	(4.2)	1.1	0.3–4.1

*Shaving from barber*						
No	17	(26.6)	100	(38.5)	1.0	-
Yes	47	(73.4)	160	(61.5)	1.7	0.9–3.2

*Frequency of going to barber*						
Never	17	(26.6)	100	(38.5)	1.0	-
Daily	17	(26.6)	65	(25.0)	1.5	0.7–3.2
Once a week	19	(29.7)	59	(22.7)	1.9	0.9–3.9
More than once a week	11	(17.2)	36	(13.8)	1.8	0.8–4.2

*Death of a family member due to liver disease*						
No	55	(85.9)	24.8	(95.4)	1.0	-
Yes	9	(14.1)	12	(4.6)	3.4	1.4–8.4

*Lived with jaundiced family member*						
No	38	(59.4)	190	(73.1)	1.0	-
Yes	26	(40.6)	70	(26.9)	1.9	1.0–3.4

*History of sexual contact*						
No history of sexual contact	24	(37.5)	130	(50.0)	1.0	-
Sexual contact with ≥ 1 partners	40	(62.5)	130	(50.0)	1.6	0.9–3.0

• Commercial Sex worker ^†^Odds ratio ^‡^Confidence Interval

•^χ ^Who provides services like laborer, driver, policeman, factory worker etc.

**Table 3:** Multivariate logistic regression model of risk factors associated with hepatitis B virus infection in asymptomatic volunteer blood donors in a case-control study, Karachi, Pakistan.

	Adjusted odds ratio	95 % confidence interval
*History of dental treatment*		
Never had dental treatment	1.0	-
Treated by dentist	1.6	0.7 – 3.6
Treated by un-qualified provider	9.8	2.1 – 46.1
		
*Number of therapeutic injection received in last five years*		
0	1.0	-
1–5	3.3	1.1 – 9.6
> 5	4.2	1.4 – 12.7
		
*Type of syringe used for the last injection*		
Never had injection	1.0	-
Plastic	1.8	0.5 – 6.4
Glass	9.4	2.6 – 34.3
		
*Injury resulted in bleeding during facial shaving from barber*		
No	1.0	-
Yes	2.3	1.1 – 4.8
